# Editorial: Pharmacology of BPSD (Behavioral and Psychological Symptoms of Dementia)

**DOI:** 10.3389/fphar.2021.704421

**Published:** 2021-06-15

**Authors:** Lydia Giménez-Llort, Björn Johansson

**Affiliations:** ^1^ Department of Psychiatry and Forensic Medicine, School of Medicine, Universitat Autònoma de Barcelona, Barcelona, Spain; ^2^ Institut de Neurociències, Universitat Autònoma de Barcelona, Barcelona, Spain; ^3^ Department of Clinical Neuroscience, Karolinska Institutet, Stockholm, Sweden; ^4^ Department of Molecular Medicine and Surgery, Karolinska Institutet, Stockholm, Sweden; ^5^ Theme Inflammation and Aging, Karolinska University Hospital, Stockholm, Sweden

**Keywords:** behavioral symptoms, dementia, drug discovery, interdisciplinary research, neuropsychiatric symptoms, pharmacology

This Research Topic started out on a sombre note. Despite being common in older adults and affecting even some young people, awareness is limited about neuropsychiatric symptoms (NPS) in dementia, the so-called BPSD, even among health professionals, and only a few drugs registered for them. Around 2018, several pharmaceutical companies stopped or downsized neuroscience research divisions oriented toward Alzheimer’s disease (AD), some of which had drug candidates of potential interest for BPSD. Antipsychotics had fallen out of favor in many locations, as there were reports of elevated mortality that restricted their use. Nonpharmacological treatments usually mandated by law or guidelines were often given limited resources; there was limited agreement about the optimal nonpharmacological treatment and scarce research on them. To address this situation, this Research Topic has involved multiple disciplines ranging from clinics to basic research. The idea is to move away from an abstract view of BPSD toward a detailed understanding and optimized therapeutic interventions. This Research Topic presents 31 papers (one is this Editorial and one is a correction); 139 authors contributed to these.

## Antipsychotics, Old and New

The contributions by Calsolaro et al. and Magierski et al. start out by listing the symptoms counted as BPSD and notice that they may occur at any stage of dementia, the typical time profile depending on the type of dementia. The authors go on to present the more conventional drug treatments for BPSD such as dopamine receptor antagonists and move on to newer drugs. The contribution by Magierski et al. also includes a section on nonpharmacological treatments. The review by Yunusa and El Helou deals with the single antipsychotic, risperidone, approved for BPSD (one component of it, aggression) in many countries. It brings up statistically significant effectiveness of this antipsychotic against BPSD in some situations and lists side-effects and regulatory differences among countries. Ohno et al. point out that antipsychotic drugs can have extrapyramidal side effects, including Parkinsonian symptoms, also when used in elderly with dementia. These authors discuss drug choice and combination strategies, as cholinesterase inhibitors and antidepressants that are 5-HT reuptake inhibitors may potentiate EPS whereas NMDA receptor antagonist memantine and adrenergic and 5-HT receptor antagonist mirtazapine reduce EPS. These authors go into some detail on modulation of EPS by 5-HT receptor subtypes. The contribution by Yunusa et al. highlights encouraging preliminary results on a newer antipsychotic, pimavanserin, for the treatment of dementia-related psychosis (already FDA-approved in U.S. for Parkinson-related psychosis), the first antipsychotic with little antagonism of adrenergic, dopaminergic, histaminergic and muscarinic receptors, but significant interactions with 5-HT receptors.

## New Looks at Traditional Medicines

Looking back in time but also into the future, the papers by Park et al. and Fu et al. deal with herbal medicines that have traditionally been, and still are, given to old people for i.a. cognitive and depressive symptoms. Both explore how these (and potentially other traditional drugs) can be researched using modern methods and formulations improved. Interestingly, both drugs described have statistically significant effects in some of the assays used. The systematic review by Fu et al. of a medicine traditionally used in the old people found an improvement in preclinical scores for cognitive functioning and mood. Possible mechanisms for Kaixinsan (KXS) were found to be antioxidant, anti-inflammatory and antiapoptotic activity, neuroprotection and synapse protection. suggesting that KXS might be considered for depressive symptoms in dementia acting through multiple mechanisms. The study of PSM-04, derived from a natural medicine, by Park et al. addresses mechanisms of another drug that is traditionally used in elderly with cognitive or depressive problems, addressing its mechanisms. In preclinical models, PSM-04 had significant neuroprotective effects against neurotoxicity induced by L-glutamate or oligomeric Aβ and decreased oxidative stress induced by H_2_O_2_. The drug also reduced cognitive impairment and decreased amyloid deposition in a mouse model. This paper points to the interesting possibility that the mood improvement by PSM-04 (and related drugs) may be mediated (in part) by reduction in amyloid deposits. Both Park et al. and Fu et al. mention neuroprotection and reduction in oxidative stress, suggesting mechanisms that may be common to both KXS and PSM-04 and to both cognitive and depressive symptoms.

## Advances in Methodology and IT

The contribution of Lin and Lane deals specifically with glutamate-related mechanisms. It starts from memantine, the NMDA receptor antagonist used in AD, and notices evidence that both under- and overactivity can be detrimental, and suggest that “precision medicine,” specifically tailoring treatment to each patient using NMDA-related biomarkers, can be helpful. Somewhat less high-tech, but possibly important technology development is the blood concentration measurements of AChE inhibitors (AChE-I) by Ortner et al. AChE-I are used for the treatment of cognitive symptoms of dementia but also BPSD. Ortner et al. find that serum concentrations below the recommended range in about two thirds of the patients and suggest that therapeutic drug monitoring might help to identify the cause of poor clinical response of cognition and behavioral and psychological symptoms in patients. This could be especially relevant to BPSD, as there is some evidence that relatively higher doses of AChE-I might be needed for BPSD than for cognitive impairments. IT is the main theme of two contributions, Piau et al. and Husebo et al. The contribution by Piau et al. focuses on digital biomarkers and points to an unmet need to monitor nature, frequency, severity, impact, progression, and response to treatment of BPSDs after the initial assessment. to reevaluate therapeutic strategies more quickly and, in some cases, to treat earlier, when symptoms are still amenable to therapeutic solutions or even prevention. These authors suggest digital biomarkers are monitoring more than diagnostic tools. In addition to the implications for clinical care, several ways to use digital biomarkers for more effective pharmaceutical research are suggested. Husebo et al. find that technology can often pick up behaviors of BPSD such as sleep disturbances, agitation and wandering, and may be well accepted. These authors also propose a framework for sustainable ethical innovation in healthcare technology. Advancements in methodology of a different kind, i.e., design of clinical trials, are proposed in Hulshof et al. This study indicates that resources have been wasted in clinical trials of antipsychotics for neuropsychiatric symptoms of dementia, because sample sizes were either too small or unnecessarily large. The article suggests ways to improve clinical trials in the BPSD area both during study design and when findings are later reported. Interesting methodology developments in the use of animal models are dealt with in the contributions by Torres-Lista et al. and Pifferi et al. A string of publications from the first-mentioned lab shows that BPSD can be modeled in mice. In their mouse model, the benefits of risperidone were limited, both at cognitive and BPSD-like level, and there was early and long-lasting mortality risk. Since mortality from an antipsychotic is reproduced in this mouse model, it might be possible to address antipsychotic toxicity using this animal model. A fresh approach to BPSD may be the one of Pifferi et al. Here, it is suggested that the gray mouse lemur, a primate, is a more translatable animal model of dementia and BPSD. Interestingly, effectiveness of approved cognitive enhancers such as acetylcholinesterase inhibitors or N-methyl-D-aspartate antagonists is demonstrated in sleep–deprived animals. Knowledge of similarities between age-related symptoms in the lemur with BPSD in humans might help understand and treat BPSD.

## Focus on Depressive and Other Specific Symptoms

Several contributions deal with the depressive symptoms that commonly co-exist with or precede cognitive impairments in dementia disorders, and with the related but distinct problem of apathy in dementia. The contribution by Cassano et al. begins by noticing that the question if depression is a prodromal symptom preceding cognitive deficits or an independent risk factor for AD is still unclear, although a connection between depressive disorders and AD is widely recognized. Moreover, there is growing evidence reporting that conventional antidepressants are not effective in depression associated with AD and, therefore, there is an urgent need to understand the neurobiological mechanism underlying the resistance to the antidepressants. The paper by Linnemann and Lang explores possible mechanistic relationships between late-life depression and dementia, both of which are common in old age and often occur together. The fact that depression often comes with cognitive impairment and dementia often presents with depressive symptoms is a challenge. However, more than six several pathophysiological substrates are proposed to explain the link between late-life depression and dementia. The Systematic Review of Wiels et al. suggests that depressive symptoms and dementia have common risk factors, that depressive symptoms being a prodromal symptom of dementia and/or depression being a risk factor for dementia. To the editors, it seems that all these causal relationships may be operating at the same time. Since clinical diagnostic criteria were usually used, not biomarkers, different pathophysiologies might be operative in different patients. The contributions on apathy by Bogdan et al. and Theleritis et al. point out the lack of drive in many patients with dementia. They notice that apathy in dementia may have separate pathways from depression and points to several possible targets for drug treatment in apathy, although approved agents for apathy are still missing. The authors suggest early treatment is important, as apathy may have a negative impact on the disease progression. They suggest that optimizing treatment duration and samples sizes may improve future clinical trials. Besides Bogdan et al., Gottesman and Stern deal with the question of BPSD and rate of cognitive decline. They make the interesting point that BPSD is positively correlated with the rate of decline in AD and suggest that the presentation and course of AD is highly heterogenous with BPSD contributing to heterogeneity. The HIDA axis is almost the antithesis of depressive symptoms: Hyperactivity–Impulsivity–Irritiability–Disinhibition–Aggression–Agitation. Keszycki et al. argue that some symptoms in the HIDA axis do not respond adequately to nonpharmacological treatments, necessitating adjunct pharmacological intervention.

## Mechanisms of BPSD and Possible Connections With Other Disorders

Ng et al. sort the diverse symptoms of BPSD into subsyndromes and notice that they may arrive early in AD, even before the onset of cognitive impairment. They point to metabolic dysfunction in specific brain areas and networks in the different BPSD. They find that neuropsychiatric symptoms are associated with poorer outcomes in cognition and function and discuss opportunities of intervention. The study by Liu et al. addresses whether anticholinergic activity of drugs is associated with risk of dementia and BPSD. Drugs with anticholinergic activity are strongly suspected to increase risk of dementia. However, this study found limited evidence for such relationships; nevertheless, with high anticholinergic burden the relationship became apparent. Thus, there is still limited consensus connecting dementia with anticholinergic activity; however, toward the end of their paper, these authors give suggestions to design more definitive studies in the future. Some aids for drug review in dementia discommend tricyclic antidepressants due to their anticholinergic effects (Religa et al., 2021, in press). Nevertheless, Hessmann et al., in their contribution, found that patients being diagnosed with dementia frequently are prescribed tricyclic antidepressants. In many locations, the tricyclics would be deprescribed during regular medication reviews. However, considering the contribution of Liu et al. suggesting limited increase in dementia risk by a modest exposure to anticholinergic drugs, and since tricyclics are sometimes prescribed after nonresponse to other antidepressants, could this prescription be warranted in some instances? Disorders of the urinary system are common in geriatric patients with dementia. Many such patients have reduced kidney function, although the only sign of it may be abnormal markers such as creatinine. The contribution of Simões e Silva et al. explores the connection between chronic kidney disease and neuropsychiatric disorders, among which depression is common. There is some evidence for increased cerebrovascular disease from uremic toxins, but the authors find evidence for bidirectional cross-talk between brain and kidney through mechanisms including inflammatory cytokines and the renin-angiotensin system. There is evidence for involvement of the kynurenine pathway, an alternative pathway of tyrosine metabolism, in neuropsychiatric symptoms as well as AD progression and neurodegeneration. In some tissues with AD-type changes, overactivity of the kynurenine pathway has been demonstrated, which is likely to increase levels of certain neurotoxic metabolites. Against this background, Fertan et al. tested a whether a novel inhibitor of this pathway could improve cognitive and behavioral changes in a mouse model of AD. DWG-1036 showed improvements in in memory and behavior in several but not all assays used. Could the kynurenine pathway be one that connects BPSD and memory loss? The contribution of Tarasov et al. is relevant because of a recent surge in publications implicating a role of astrocytes in AD and other neurodegeneration (Verkhratsky et al., 2019). They review evidence for alterations of astrocytes also in schizophrenia in which their role may be neuroinflamation and an indirect influence on dopamine neurons *via* NMDA receptors. A state of hyperactive dopamine projections in the mesolimbic system and reduced dopamine projections in the mesocortical system in schizophrenia is mentioned. Could it be that dopamine-related mechanisms of Tarasov et al. be a cause of hypometabolism in frontal cortical areas in AD with psychosis reviewed in the contribution by Ng et al.? Autism and BPSD are seldom considered together, but the contribution by Eissa et al. compares these two conditions side by side. They identify some commonalities in symptoms and mechanistic roles of inflammation, histamine, amyloid precursor protein (APP) and changes in white matter. The experimental study of Scuteri et al. points to the possibility that BPSD can be a manifestation of pain, a common co-morbidity that these patients can have difficulty to express. It was found that formalin-induced behavioral pattern in older mice was different and suggests a possible animal model of pain in the elderly.

## Final Notes

Finalizing this Research Topic, we are now optimistic. A few trends can be discerned: Antipsychotics, even those that are strong blockers of dopamine receptors, have not been written off from treatment of BPSD. Side effects of antipsychotics may at times be effectively dealt with, as described here. New antipsychotics with different receptor binding profile may have a better benefit to risk ratio. Improvements in methodology such as IT, biomarkers, drug concentration measurements and study design will optimize the use of existing drugs and speed discovery of new drugs. Traditional medicines are still of interest, and new technologies may make them more effective and useful.

**FIGURE 1:**
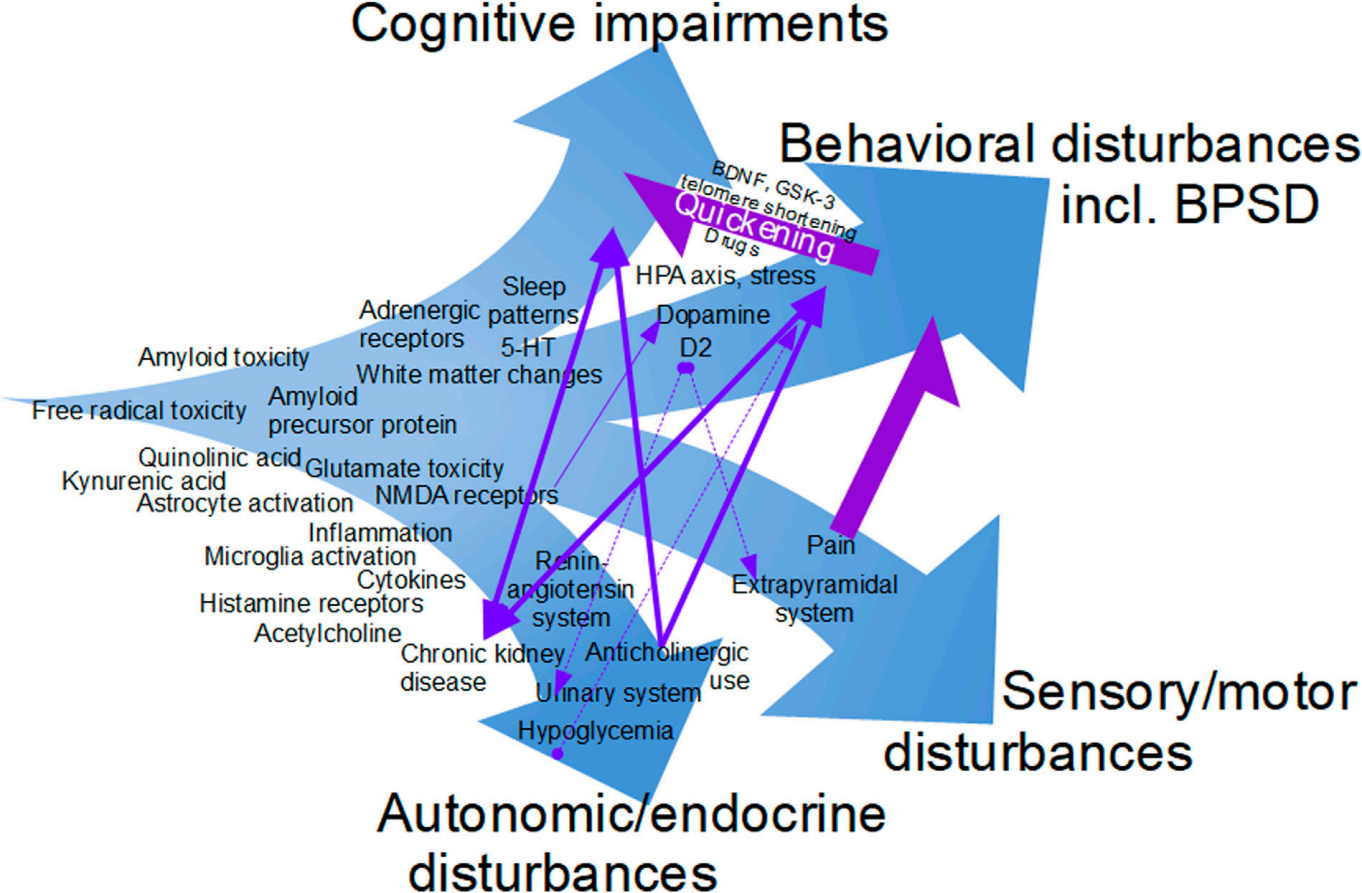
The multiple facets of the dementias, as gleaned from the contributions to this Research Topic. Relationships among cognitive impairments, behavioral problems and co-morbidities such as pain and disorders of the urinary system. This figure is work in progress, as this version is not a complete summary of all mechanisms mentioned in the contributions or all suggestions received from the authors.

The new disease classification ICD-11, being introduced internationally, associates dementias with a specific nervous system disease process and, if needed, BPSD or a specific symptom of BPSD (World Health Organization, 2018). The present Research Topic outlines many mechanistic pathways, containing potential drug targets, for BPSD but also cognitive impairments and CNS disease processes. Candidate drugs against AD are often divided in three groups (Cummings et al., 2020), the larger “disease-modifying” group, and the groups whose purpose is cognitive enhancement or controlling neuropsychiatric symptoms. However, it has been noticed that some agents may belong to more than one group (Cummings et al., 2020), and several drugs and mechanisms mentioned in this Research Topic can reasonably be placed in more than one group. Several of the contributions touch upon possibly overlapping neural/mechanistic pathways with comorbidities (pain, urinary system disorders). Bladder dysfunction, for which drugs with anticholinergic activity are often prescribed, may be an integrated part of neurodegenerative disorders or a consequence of treatment prescribed (Winge, 2015). Perhaps altered behavior patterns during micturition might be considered a specific Behavioral and Psychological Symptom of Dementia? The evidence that BPSD are associated with quickened cognitive decline is of clear interest to pharmacologists, primarily because some drugs used in BPSD might quicken cognitive decline (see Gottesman and Stern). However, an interesting possibility is that optimized treatment of BPSD could reduce cognitive decline, as there is very recent evidence that that the abovementioned antipsychotic pimavanserin might be disease-modifying in AD (Yuede et al., 2021) as well as effective against neuropsychiatric symptoms. Park et al. presents evidence for an anti-amyloid effect of a natural medicine used i.a. for depressive symptoms in the elderly, which might be an indication that amyloid contributes to such symptoms. Figure 1 tries to place mechanisms mentioned in this Research Topic in the context of the multiple clinical manifestations of the dementias.

We feel that the multidisciplinary approach taken in this Research Topic has been successful and will quicken progress in the BPSD treatment. *For natural reasons, many therapies described here are of an experimental nature, so always check with law and local rules and guidelines before making clinical decisions.*

## References

[B1] Cummings J. Lee G. Ritter A. Sabbagh M. Zhong K. (2020). Alzheimer's Disease Drug Development Pipeline: 2020. Alzheimer's Demen. Translational Res. Clin. Interventions 6, e12050. 10.1002/trc2.12050 PMC7364858 32695874

[B2] Religa D. Wieczorowska-Tobis K. Johansson B. (2021). “Review of Medication in Patients with Dementia,” in Management of Patients with Dementia: The Role of the Physician. Editors Frederiksen, Kristian Steen Waldemar Gunhild (Cham (Switzerland): Springer) (in press).

[B3] Verkhratsky A. Parpura V. Rodriguez-Arellano J. J. Zorec R. (2019). “Astroglia in Alzheimer’s Disease. Chapter 11,” in Neuroglia in Neurodegenerative Diseases, Advances in Experimental Medicine and Biology 1175. Editors (Springer Nature Singapore Pte Ltd). 10.1007/978-981-13-9913-8 PMC7266005 31583592

[B4] Winge K. (2015). Lower Urinary Tract Dysfunction in Patients with Parkinsonism and Other Neurodegenerative Disorders. Handbook Clin. Neurol. 130 (3rd series) 2015 Elsevier B.V. 10.1016/B978-0-444-63247-0.00019-5 26003253

[B5] World Health Organization (2018). ICD-11 International Classification of Diseases 11th Revision. Available at: https://icd.who.int/en (Accessed April 30, 2021).

[B6] Yuede C. M. Wallace C. E. Davis T. A. Gardiner W. D. Hettinger J. C. Edwards H. M. (2021). Pimavanserin, a 5HT 2A Receptor Inverse Agonist, Rapidly Suppresses Aβ Production and Related Pathology in a Mouse Model of Alzheimer's Disease. J. Neurochem. 156 (5), 658–673. Epub 2021 Jan 10. 10.1111/jnc.15260 33278025 PMC7946332

